# A *Populus TIR1* gene family survey reveals differential expression patterns and responses to 1-naphthaleneacetic acid and stress treatments

**DOI:** 10.3389/fpls.2015.00719

**Published:** 2015-09-10

**Authors:** Wenbo Shu, Yingli Liu, Yinghua Guo, Houjun Zhou, Jin Zhang, Shutang Zhao, Mengzhu Lu

**Affiliations:** ^1^Co-Innovation Center for Sustainable Forestry in Southern China, Nanjing Forestry UniversityNanjing, China; ^2^State Key Laboratory of Tree Genetics and Breeding, Research Institute of Forestry, Chinese Academy of ForestryBeijing, China

**Keywords:** *Populus*, FBL family, phylogenetic analysis, expression, NAA, stress responses

## Abstract

The plant hormone auxin is a central regulator of plant growth. TRANSPORT INHIBITOR RESPONSE 1/AUXIN SIGNALING F-BOX (TIR1/AFB) is a component of the E3 ubiquitin ligase complex SCF^TIR1/AFB^ and acts as an auxin co-receptor for nuclear auxin signaling. The SCF^TIR1/AFB^-proteasome machinery plays a central regulatory role in development-related gene transcription. *Populus trichocarpa*, as a model tree, has a unique fast-growth trait to which auxin signaling may contribute. However, no systematic analyses of the genome organization, gene structure, and expression of TIR1-like genes have been undertaken in this woody model plant. In this study, we identified a total of eight *TIR1* genes in the *Populus* genome that are phylogenetically clustered into four subgroups, *PtrFBL1/PtrFBL2, PtrFBL3/PtrFBL4, PtrFBL5/PtrFBL6*, and *PtrFBL7/PtrFBL8*, representing four paralogous pairs. In addition, the gene structure and motif composition were relatively conserved in each paralogous pair and all of the *PtrFBL* members were localized in the nucleus. Different sets of *PtrFBLs* were strongly expressed in the leaves, stems, roots, cambial zones, and immature xylem of *Populus*. Interestingly, *PtrFBL1* and *7* were expressed mainly in vascular and cambial tissues, respectively, indicating their potential but different roles in wood formation. Furthermore, *Populus FBLs* responded differentially upon exposure to various stresses. Finally, over-expression studies indicated a role of FBL1 in poplar stem growth and response to drought stress. Collectively, these observations lay the foundation for further investigations into the potential roles of *PtrFBL* genes in tree growth and development.

## Introduction

Plant growth and development are largely influenced by the surrounding environment, which is mediated by different phytohormones through signaling pathways. As a central regulator involved in nearly all aspects of plant development, auxin is synthesized by various plant tissues (Lau et al., 2009) and transported long-distance through vascular tissues or from cell to cell across membranes (Kramer and Bennett, 2006) to contribute to the robustness of the auxin signaling network (Peer, 2013). Within minutes, auxin can rapidly alter the expression of hundreds of genes, (Chapman and Estelle, 2009) which were classified into three major families: *SMALL AUXIN-UP RNA* (*SAUR*), *GH3*, and *AUXIN*/*INDOLE-3-ACETIC ACID* (*Aux/IAA*) genes. The *SAUR* genes encode unstable transcripts (Chapman and Estelle, 2009); the *GH3* family encodes enzymes that catalyze the conjugation of IAA with an amino acid to yield an inactive storage form of IAA (Ludwig-Müller, 2011); and the *Aux/IAA* family plays a central role in auxin signal transduction that regulates the expression of auxin-responsive genes (Remington et al., 2004), which are involved in growth, development, and responses to abiotic stresses such as salt, drought, and temperature (Salehin et al., 2015).

The diversity in auxin perception is a key factor leading to the great variety of auxin responses (Woodward and Bartel, 2005). Three receptor/co-receptor complexes involved in auxin signaling have been identified: two inside of the cell [SCF^TRANSPORT INHIBITOR RESPONSE 1/AUXIN SIGNALING F−BOX (TI R1/AFB)^ and SCF^S−PHASE KINASE−ASSOCIATED PROTEIN 2A (SKP 2A)^] and one at the cell periphery, Auxin Binding Protein 1 (ABP1; Salehin et al., 2015). TIR1/AFB-Aux/IAA, the best characterized receptor system, regulates transcription of downstream auxin-responsive genes in the nucleus (Calderón Villalobos et al., 2012), while newly identified auxin receptors SKP2A and ABP1 mainly repress cell division during the cell cycle and subcellular protein trafficking, respectively (Jurado et al., 2008; Robert et al., 2010).

Nuclear auxin signaling involves TIR1/AFBs that is a component of the E3 ubiquitin ligase complex SCF^TIR1^ (Gagne et al., 2002) and acts as an auxin co-receptor (Dharmasiri et al., 2005a,b). Auxin enhances the TIR1-Aux/IAA interaction by filling the bottom of a hydrophobic pocket in TIR1, which changes the properties of SCF^TIR1^ (Yu et al., 2013). The SCF^TIR1/AFB^-proteasome machinery causes the proteolytic turnover of Aux/IAAs, thereby releasing ARFs and controlling the specificity of the downstream genes' transcription (Calderón Villalobos et al., 2012; Yu et al., 2013). Therefore, TIR1/AFB is the trigger that activates the auxin pathway.

Six *Arabidopsis* TIR1/AFB receptors, interacting with 29 Aux/IAA and 23 ARF combinations, modulate precisely the specific gene expression profile in response to auxin signaling (Salehin et al., 2015). TIR1 and AFB2 are positive regulators of auxin signaling (Parry et al., 2009), while AFB4, as a negative regulator, is epistatic to TIR1 and AFB2 (Greenham et al., 2011; Hu et al., 2012). TIR1 and AFB2 play more important roles during seedling development and root responses to the environment than either AFB1 or AFB3 (Parry et al., 2009). AFB3 has a unique role in the nitrate response of roots (Vidal et al., 2010), and AFB2 and AFB3 also have important roles in seed coat development (Locascio et al., 2014). AFB5 is part of an auxin receptor complex with a higher binding affinity for picloram (4-amino-3, 5, 6-trichloropicolinic acid) and specifically recognizes synthetic picolinate auxins (Walsh et al., 2006). TIR1/AFBs show different affinities for given Aux/IAAs (Parry et al., 2009; Calderón Villalobos et al., 2012) and the half-lives are significantly different among Aux/IAAs (Dreher et al., 2006). For a given Aux/IAA, TIR1, and AFB2 confer a more rapid degradation than AFB1 and AFB3 do (Havens et al., 2012). These results suggest different roles for TIR1/AFB in the regulation of auxin signaling, temporally, and spatially, under different stresses, such as salt (Chen et al., 2015) and drought (Guerra et al., 2015).

Woody plants have unique biological processes that are absent in model herbivorous plants, like *Arabidopsis*. For instance, continuous cylinder-like meristem is reactivated each year in woody plants (Fuchs et al., 2010). How the auxin signaling functions during these unique processes in woody plants remains to be elucidated. The recent sequencing of the *Populus trichocarpa* genome (Tuskan et al., 2006) has provided an opportunity to study the families of auxin signaling-related genes, such as *Aux/IAA, ARF* (Kalluri et al., 2007), *WUSCHEL* (*WUS*) (Liu et al., 2014a), and *PIN-FORMED* (*PIN*) (Liu et al., 2014b), laying the foundation for further investigations into the roles of auxin signaling in woody plant development. In this study, using *Arabidopsis AtTIR1/AFB*, we have identified *PtrFBLs* in the *P. trichocarpa* genome, and determined their structure, phylogenetic relationships and expression patterns. Eight *PtrFBL* members, as well as their expression profiles in different tissues and their responses under 1-naphthaleneacetic acid (NAA), heat, and drought treatments, are presented.

## Materials and methods

### Bioinformatics analysis

Published *AtTIR1/AFB* and *PtrFBL* gene sequences (Parry et al., 2009) were retrieved and used as queries in BLAST searches against the *Arabidopsis* and *Populus* genome databases (http://phytozome.jgi.doe.gov/pz/portal.html). Multiple full-length sequences of AtTIR1/AFB and PtrFBL proteins were aligned using the CLUSTALX 2.0 software (Larkin et al., 2007). Un-rooted phylogenetic trees were constructed with MEGA 5 using the neighbor-joining method, with 1000 bootstrap replicates (Tamura et al., 2011). WoLF PSORT (http://www.genscript.com/psort/wolf_psort.html) was used to predict protein subcellular localizations. The isoelectric point and molecular weight were estimated using the Compute pI/Mw tool from ExPASy (http://web.expasy.org/compute_pi). A search for duplicated genes was performed using the Plant Genome Duplication Database (PGDD; http://chibba.agtec.uga.edu/duplication/). The chromosomal locations of the *PtrFBL* genes were determined using the *Populus* genome browser (http://phytozome.jgi.doe.gov/pz/portal.html#!search?show=KEYWORD&method=Org_Ptrichocarpa). The exon and intron structures of individual *TIR1/AFB* genes were illustrated using the Gene Structure Display Server (GSDS; http://gsds.cbi.pku.edu.cn/) (Hu et al., 2015) by aligning the cDNA sequences with the corresponding genomic DNA sequences.

Functional motifs or domains of TIR1/AFB and PtrFBL protein sequences were analyzed using PROSITE and the Conserved Domain database. MEME (http://meme.nbcr.net/meme/) (Bailey et al., 2009) was used to identify motifs in candidate sequences with the following parameters: number of repetitions = any, maximum number of motifs = 20 and optimum motif widths constrained to from 30 to 70 residues.

### Plasmids and constructs

The coding sequences of *PtrFBL1, 2, 3, 4, 5, 6, 7*, and *8*, without stop codons, were amplified from the cDNA of hybrid poplar 84K (*Populus alba* × *Populus glandulosa*) and inserted into pEarleyGate101 (ABRC stock DB3-683) to produce the 35S::*PtrFBL1*-YFP, 35S::*PtrFBL2*-YFP, 35S::*PtrFBL3*-YFP, 35S::*PtrFBL4*-YFP, 35S::*PtrFBL5*-YFP, 35S::*PtrFBL6*-YFP, 35S::*PtrFBL7*-YFP, and 35S::*PtrFBL8*-YFP constructs, respectively, using the Gateway cloning system (Invitrogen). The approximately 2.5 kb 5′-UTR fragments of *PtrFBL1, PtrFBL4, PtrFBL5*, and *PtrFBL7* were amplified from the genomic DNA of *P. trichocarpa* Torr. The primer sequences for the promoters are listed in Table S1. The promoter fragments were then inserted into pDNOR222.1 and subcloned into pMDC164 to produce *P*_*PtrFBL*1_::GUS, *P*_*PtrFBL*4_::GUS, *P*_*PtrFBL*5_::GUS, and *P*_*PtrFBL*7_::GUS constructs using the Gateway cloning system (Invitrogen). The coding sequence of *PtrFBL1* was amplified from the cDNA of 84K, cloned into pDNOR222.1 and sequenced. *PtrFBL1* cDNA was further cloned into pMDC32 to produce 35S::*PtrFBL1* constructs for transformation into poplar 84K. The transgenic poplar plants containing the auxin responsive promoter DR5 were obtained previously (Liu et al., 2014a). At least five independent transgenic lines were used for further analyses.

### Plant cultivation and transformation

Tobacco (*Nicotiana benthamiana*) plants were cultivated under short-day photoperiod conditions (16 h light/8 h dark). When the *Agrobacterial* culture reached the stationary growth phase at 28°C with agitation, cells were collected and resuspended in infiltration buffer (100 μM acetosyringone in 10 mM MgCl_2_) to OD_600_ = 0.8. The leaves of 2-month-old tobacco seedlings expressing 35S::*PtrFBLs*-YFP were used for infiltration. The transient expression in lower leaf epidermal cells was performed as described by Liu et al. (2014a). After 5 to 7 days, the leaves were immersed in 50 μM 4′, 6-diamidino-2-phenylindole (DAPI) for 60 min for a subcellular localization analysis. Fluorescence was observed using an UltraVIEW VoX 3D Live Cell Imaging System (PerkinElmer, USA). For imaging YFP and DAPI fluorescence, 488 and 405-nm excitations were used, respectively.

Hybrid poplar (*P*. *alba* × *P*. *glandulosa*) clone 84K was used for stable transformations (Liu et al., 2014a). Histochemical GUS staining was performed by incubating 2-week-old seedlings and 4-week-old stem sections in 90% cold acetone. Each sample was washed three times with a staining buffer containing 50 mM sodium phosphate (pH 7.0), 2 mM potassium ferrocyanide, 2 mM potassium ferricyanide, 10 mM EDTA, and 0.2% (v/v) Triton X-100 on ice. The samples were then transferred into the staining solution [staining buffer with 20% (v/v) methanol and 1 mM X-Gluc] and slowly subjected to vacuum infiltration, which penetrated the tissue with the staining solution. After a 12 h incubation at 37°C with 70 rpm gentle agitation, the samples were rinsed in 70% ethanol for visual observation and microscopy.

### RNA isolation, semi-quantitative RT-PCR and qRT-PCR

Total RNA was extracted from roots, leaves and young stems collected from young seedlings, cambial zones and immature xylem obtained by peeling the bark of 5-year-old poplar trees (Du and Groover, 2010) using the RNeasy Plant Mini Kit and treated with RNase-free DNase I (Qiagen, Hilden, Germany). Additionally, total RNA was extracted from hormone- and stress-treated seedlings collected at different times. First-strand cDNA synthesis was carried out with approximately 3 μg RNA using the Superscript III reverse transcription kit (Life Technologies, Carlsbad, CA, USA) according to the manufacturer's instruction. Specific 20–25 bp long RT-PCR primers with melting temperatures of 58–60°Cand target lengths of 150–250 bp were designed using Primer 3 software (http://frodo.wi.mit.edu/primer3/input.htm) (Table S1). The amplified fragments were confirmed using agarose gel electrophoresis. Real-time qRT-PCR was performed using the SYBR Premix Ex Taq II Kit (TaKaRa Dalian, Dalian, China) on a Roche LightCycle 480 Real-Time PCR System (Roche Applied Science, Germany) according to the manufacturer's instructions. Reactions were prepared in a total volume of 20 μl containing 10 μl of 2 × SYBR Premix, 2 μl of cDNA templates as prepared above and 1 μl of each specific primer to a final concentration of 200 nM. The reactions were performed using the following conditions: initial denaturation step of 95°C for 30 s, followed by 40 cycles of a two-step thermal profile of denaturation at 95°C for 10 s and annealing/extension at 60°C for 34 s. To verify the specificity of each primer pair, a melting curve analysis was performed by increasing the temperature at a speed of 0.06°C/s (five acquisitions per 1°C) from 60 to 95°C at the end of each run. The threshold cycle values were the means of eight values from two biological repeats for each treatment with four technical replicates. The *UBQ* gene was used as an internal control.

### NAA and stress treatment

For NAA and polyethylene glycol (PEG) 6000 treatment, 4-week-old 84K seedlings were soaked in liquid Murashige and Skoog medium (pH 5.8) under a 16 h light/8 h dark regime at room temperature (mock pretreatment) for 12 h, and then supplemented with 100 μM NAA (Sigma-Aldrich, St. Louis, MO, USA) for 10 min, 20 min, 30 min, 60 min, and 360 min, and with PEG6000 (5%) for 0.5 h, 3 h, 6 h, 12 h, and 24 h. The leaves and stems were collected separately from treated seedlings at each time point and used for qRT-PCR analyses. The experiment was performed three times with four individual seedlings in each. The growth of 4-month-old over-expressing *PtrFBL1* transgenic plants were measured on three representative lines (*PtrFBL1-6, PtrFBL1-13*, and *PtrFBL1-15*) with 12 cloned plants for each. The drought treatment was performed on the above transgenic plants and non-transgenic controls by stopping watering them in greenhouse for 6 days and re-watering, the plants were checked and photographed after 15 days. For measuring relative water content (RWC) of these poplars (*PtrFBL1-6, PtrFBL1-13*, and 84K control) under the drought treatment, 3-month-old non- and transgenic plants were deprived of watering for 0, 4, and 6 days, and then the sixth leaf was detached from plants and weighed immediately to record their fresh weight (FW). The leaves were dipped in distiled water for 12 h, then blotted briefly to remove the excess water and weighed to record the turgid weight (TW). The leaves were subjected to oven at 70 °C for 24 h to determine the dry weight (DW). The RWC was calculated using the equation: RWC = (FW – DW) × 100/(TW – DW) as described previously (Negi et al., 2015). The experiments were performed with three biological replicates per treatment and the data from transgenic lines and non-transgenic controls were subjected to analysis of the variance (*F*-test, One-Way ANOVA) and the mean comparison among treatments was based on the least significant difference (LSD) test at the 5 and 1% level of significance using the package SPSS 17.0 (SPSS Inc, Chicago IL, USA).

## Results and discussion

### Characteristics of the *TIR1* gene family in *Arabidopsis* and *Populus*

We have identified eight *TIR1* homologous genes (*PtrFBL*s) in *P*. *trichocarpa* (Table S2). All of the information on these eight *PtrFBL* genes, including gene names, locus IDs, genomic position, molecular weight (Mol. Wt), lengths and numbers of introns, are listed in Table S2. The ORF length of *PtrFBL* genes varied from 1716 bp (*PtrFBL3*) to 2172 bp (*PtrFBL7*), encoding polypeptides of 571–635 amino acids (aa), with a predicted molecular weight of 64.08–70.99 kDa and a theoretical isoelectric point that ranged from 5.47 (*PtrFBL8*) to 7.35 (*PtrFBL1*) (Table S2).

**Figure 1:**
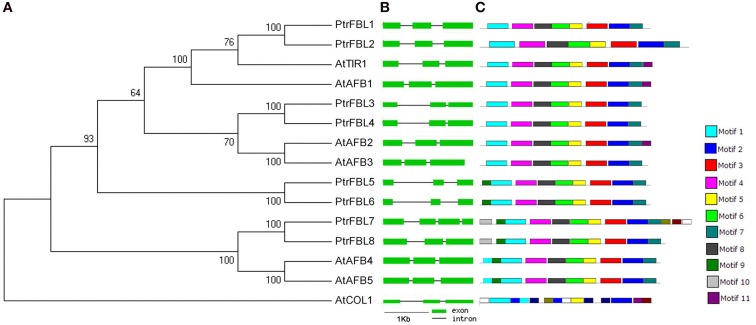
**Phylogenetic relationship, gene structures, and motif composition of ***TIR1*** genes in ***A. thaliana*** (At), and ***P. trichocarpa*** (Ptr). (A)** A phylogenetic tree was constructed using MEGA 5 by the neighbor-joining (NJ) method with 1000 bootstrap replicates. The jasmonic acid receptor CONSTANS-LIKE 1 (AtCOL1) in *Arabidopsis* was used as an outgroup. **(B)** Exon/intron positions in the TIR1 receptors. Green boxes represent exons and black lines represent introns. **(C)** Schematic representation of conserved motifs (obtained using MEME) in TIR1 proteins. Different motifs are indicated by colored boxes.

To investigate the evolutionary relationship among TIR1/AFB receptors from *Arabidopsis* and *P. trichocarpa*, a phylogenetic tree was constructed, and the TIR1 proteins were classified into four clades that include the pairs of PtrFBL1/PtrFBL2 (AtTIR1/AtAFB1), PtrFBL3/PtrFBL4 (AtAFB2/AtAFB3), PtrFBL7/PtrFBL8 (AtAFB4/AtAFB5), and PtrFBL5/PtrFBL6 (Figure 1A), suggesting that these pairs are paralogs. Each of the *Populus* paralogous pairs shared 78–90% in sequence identity, but only had 40–78% identity to *Arabidopsis* TIR1/AFBs. Surprisingly, *PtrFBL5* and *PtrFBL6* had no homologous counterparts in *Arabidopsis*. We decided it may be more accurate to ascertain the evolutionary relationships using conserved domain sequences (Marchler-Bauer et al., 2009). Therefore, we constructed the phylogenetic tree with TIR1/AFBs motif sequences from *Arabidopsis* and *Populus* using the maximum likelihood method with 1000 bootstrap replicates (Figure S1). The resulted phylogenetic tree was consistent with the one generated based on the full-length protein sequences.

In many cases, the conservation of exon/intron organization or gene structure in paralogous genes was high and could be used to reveal their evolutionary relationship (Sánchez et al., 2003). We, therefore, analyzed the exon/intron organization in the coding sequence of each *PtrFBL* in *Populus* and *TIR1/AFB* in *Arabidopsis*, which showed that each paralogous pair shared similar gene structures in terms of intron number or exon length, with the exception of *PtrFBL7* harboring one extra intron (Figure 1B, Table S2). In addition, MEME was employed to analyze motif distributions in 14 TIR1 proteins and 11 individual motifs found in *Populus* and *Arabidopsis* TIR1 receptors. Of these, motifs 3, 4, 6, 7, and 8 were found to be specific to the two species (Figure 1C, Table S3). Most of the closely related members have common motif compositions, suggesting possible functional similarities among these TIR1 proteins (Figure 1C).

Studies have found that gene duplication events are the basic contributors to evolutionary momentum (Bowers et al., 2003). The co-linear arrangements of the adjacent genes, as well as the four gene pairs (*PtrFBL1*/*PtrFBL2, PtrFBL3*/*PtrFBL4, PtrFBL5*/*PtrFBL6*, and *PtrFBL7*/*PtrFBL8*), further revealed that these paralogous pairs came from the genome duplication in *Populus* (Figure S2). The chromosomal mapping of the gene loci showed that all eight *PtrFBL* genes were distributed on 7 of 19 chromosomes, with two *PtrFBL* genes located on chromosome 2, and one each on chromosome 1, 4, 5, 11, 14, and 17 (Figure S4). *PtrFBL1*/*PtrFBL2* and *PtrFBL7*/*PtrFBL8* were distributed in the corresponding duplicated segments of the chromosomes (Tuskan et al., 2006), while *PtrFBL3*/*PtrFBL4* and *PtrFBL5*/*PtrFBL6* did not appear in the corresponding duplicated regions (Figure S3). However, only two such pairs (*AtAFB2*/*AtAFB3* and *AtAFB4*/*AtAFB5*) were found in *Arabidopsis* (Figure S5). Genome duplication events were thought to occur frequently during organismal evolution (Mehan et al., 2004). It is suggested that the *Populus* genome has experienced at least two genome-wide duplication events (eurosid and salicoid), followed by a series of chromosomal reorganizations involving reciprocal tandem/terminal fusions and translocations (Tuskan et al., 2006). Therefore, recent duplication events in *Populus* could have led to the creation of the four *PtrFBL* pairs, and the latter could have undergone intensive genome rearrangements afterwards, during which the PtrFBL3/PtrFBL4 and PtrFBL5/PtrFBL6 segments were not maintained in their corresponding duplicated regions (Figures S2, S3).

**Figure 2:**
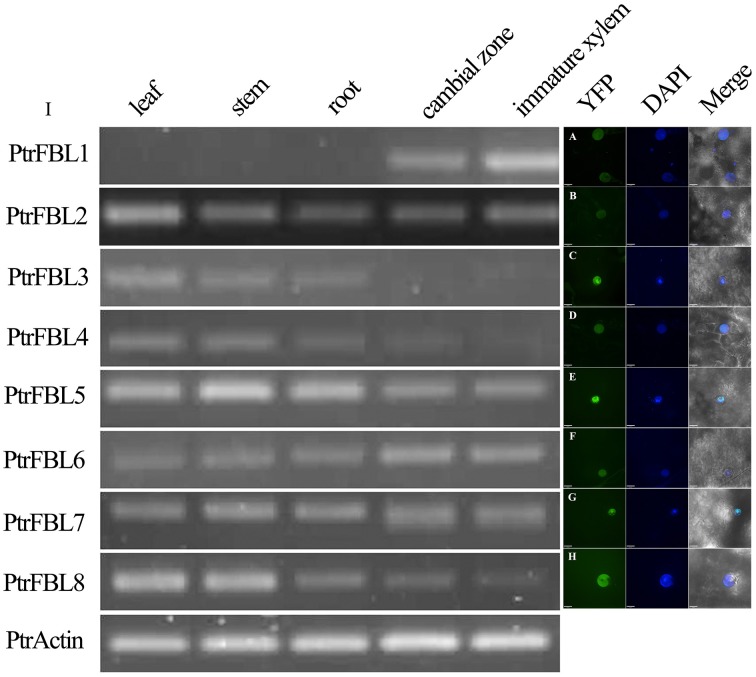
**PtrFBL protein localization (A–H) and semi-quantitative reverse-transcriptional PCR analysis of the PtrFBL expression (I)**. **(A)** PtrFBL1, **(B)** PtrFBL2, **(C)** PtrFBL3, **(D)** PtrFBL4, **(E)** PtrFBL5, **(F)** PtrFBL6, **(G)** PtrFBL7, and **(H)** PtrFBL8.

All the PtrFBLs were predicted to be localized in the nucleus using WoLF PSORT (Table S2). To confirm this, these proteins, fused with YFP at the C-terminus, were transiently expressed in tobacco leaf epidermal cells, and the fusion proteins were observed in the nucleus as indicated by co-localization with DAPI signals (Figures 2A–H). This was consistent with the nuclear localizations of TIR1/AFB proteins in *Arabidopsis* (Dharmasiri et al., 2005a; Tan et al., 2007). The conserved organelle localization of PtrFBLs implied their conserved functions, such as the regulation of the cellular auxin response.

### Expression patterns of *PtrFBLs*

The expression patterns of genes can provide useful clues to the functions of their corresponding proteins. The expression of *PtrFBL*s in the leaves, stems, roots, cambial zones and immature xylem of *Populus tomentosa* Carr. was analyzed by semi-quantitative RT-PCR (Figure 2I). *PtrFBL1* was mainly expressed in the cambial zone and immature xylem, while *PtrFBL3* and *4* were mainly expressed in the leaves, stems and roots. On the contrary, *PtrFBL2, 3*, and *8* were enriched in the leaves. The expression levels of *PtrFBL1* and *6* in the cambial zone were higher than in the other tissues, while *PtrFBL5* and *8* were found at higher levels in young stems but at lower levels in secondary vascular tissues. The *PtrFBL3*/*PtrFBL4* and *PtrFBL7*/*PtrFBL8* pairs, which had similar promoter sequences based on the phylogenetic analysis of 2.5 kb upstream sequences of the *PtrFBL* genes (Figure S4), exhibited similar expression patterns. The analysis of the RNA-seq data from different *Populus* vegetative tissues (unpublished data) further confirmed these *PtrFBLs* expression patterns (Figure S5).

**Figure 3:**
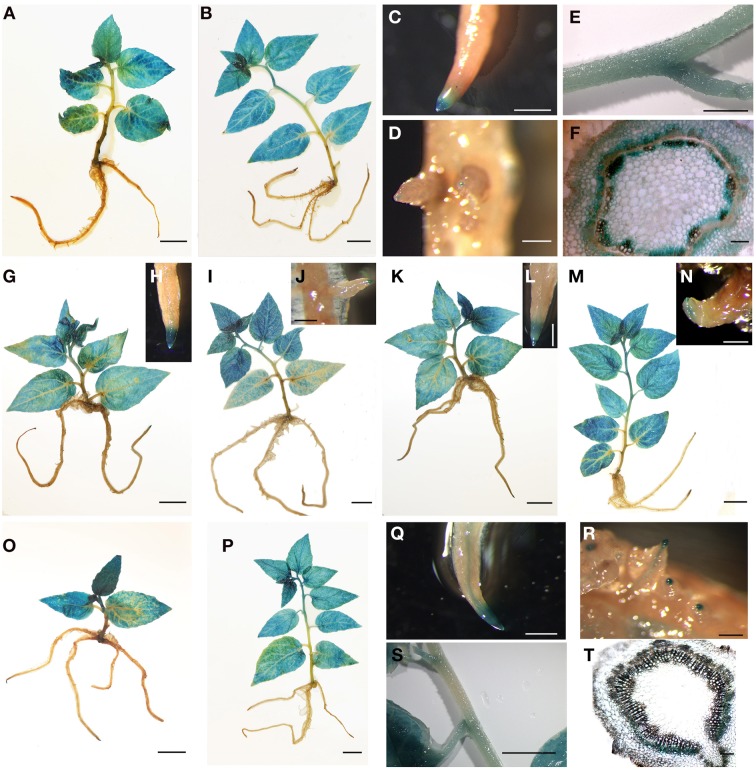
**GUS assay of transgenic poplars driven by *PtrFBL* promoters. (A–F)** *P*_*PtrFBL*1_::GUS: **(A)** 2-week-old seedling, **(B)** 4-week-old seedling, **(C)** root tip, **(D)** lateral root tip, and **(E,F)** GUS expressed mainly in stem; **(G–J)** *P*_*PtrFBL*4_::GUS: **(G)** 2-week-old seedling, **(I)** 4-week-old seedling, **(H)** root tip and **(J)** lateral root tip; **(K–N)** *P*_*PtrFBL*5_::GUS: **(K)** 2-week-old seedling, **(M)** 4-week-old seedling, **(L)** root tip, and **(N)** lateral root tip; **(O–Q)** *P*_*PtrFBL*7_::GUS: **(O)** 2-week-old seedling, **(P)** 4-week-old seedling, **(Q)** root tip and **(R)** lateral root tip; **(S,T)** GUS is expressed mainly in stems near the petiole. Bars, 1 cm **(A–C,E,G,I,K,M,O,P,S)**, 2 mm **(D,H,J,L,N,Q,R)**, 0.1 mm **(F,T)**.

To further confirm the expression patterns of the *PtrFBL* genes, four *PtrFBLs* with different expression patterns in different tissues were selected for analysis using a promoter::GUS assay. The transgenic poplars (*P. alba* × *P. glandulosa*) with *P*_*PtrFBL*1_::GUS, *P*_*PtrFBL*4_::GUS, *P*_*PtrFBL*5_::GUS and *P*_*PtrFBL*7_::GUS were obtained, and GUS assays of whole plants were performed. Similar to the expression observed in *Arabidopsis*, expression levels of these *PtrFBLs* were high in the young tissues but low in the older tissues (Figure 3). However, the expression levels varied in different organs and tissues. Except in young leaves, primary stems, roots and lateral root tips, *PtrFBL1* was found to mainly express in vascular tissues abaxial and adaxial to the cambium (Figures 3E,F), in accordance with semi-quantitative PCR results (Figure 2I). *PtrFBL7* was also observed to be highly expressed in the cambial zone (Figure 2I), but closer observations revealed the expression was restricted to the cambium in the stem sections (Figures 3S,T), in compensation to the area of vascular tissues uncovered by the *PtrFBL1* expression. However, although GUS staining of the whole plants with *P*_*PtrFBL*4_::GUS and *P*_*PtrFBL*5_::GUS confirmed the general expression patterns of these genes (Figures 3G–N), the expression was present in root tips (Figures 3H,J,L,N) and absent in root vascular tissues. In *Arabidopsis*, the AtAFB1::GUS protein was abundant throughout the *Arabidopsis* seedling, while the accumulations of AtTIR1, AtAFB2, and AtAFB3 proteins were highly restricted to growing tissues, including root tips, leaf primordia, and shoot meristems (Dharmasiri et al., 2005b; Parry et al., 2009). Therefore, poplar *PtrFBLs* exhibited more diversified expression patterns in different tissues. For instance, the differential expression of *PtrFBL1* and *PtrFBL7* was observed in secondary vascular tissues (Figure 3). In addition, these two genes may play differential roles in the maintenance of cambial activity and its differentiation into vascular tissues by mediating different auxin signaling. This phenomenon could not be observed in *Arabidopsis* because it lacks secondary vascular tissues.

### Low NAA responses of *PtrFBLs*

**Figure 4:**
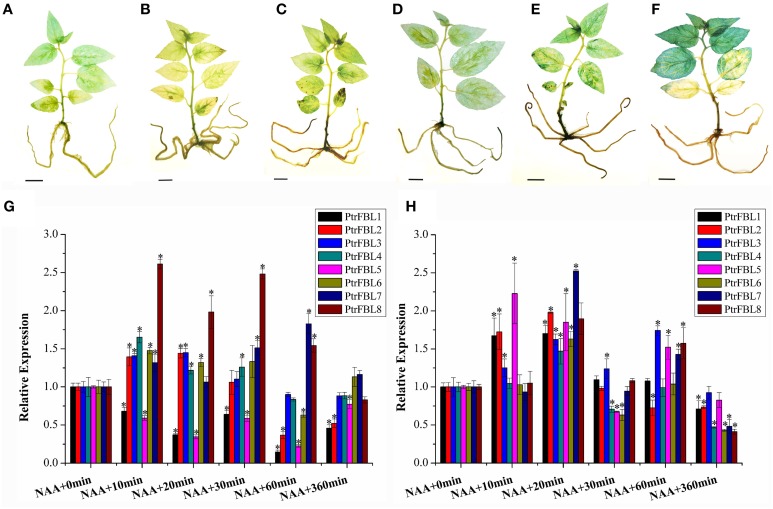
**The changes of endogenous IAA level (A–F) and PtrFBL expression (G,H) under NAA treatment. (A)** Untreated control; **(B)** NAA, treated 10 min; **(C)** NAA, treated 20 min; **(D)** NAA, treated 30 min; **(E)** NAA, treated 60 min; **(F)** NAA, treated 360 min. **(G)** Relative expression of *PtrFBLs* in 84k leaves after NAA treatments. **(H)** Relative expression of *PtrFBLs* in 84k stems after NAA treatments. Bars, 1 cm **(A–F)**, ^*^indicates significant difference at *P* < 0.05.

To reveal whether *PtrFBL* genes respond to auxin, we analyzed the expression profiles of *PtrFBLs* in poplar treated with NAA. We used poplar lines with DR5::GUS as an auxin marker (Liu et al., 2014a) to monitor the response of auxin in whole seedlings under NAA treatment (Figures 4A–F), which showed that the endogenous auxin response decreased at an early stage then increased gradually. The *PtrFBL* transcripts in general were not significantly affected by NAA treatment as quantified by qRT-PCR (Figures 4G,H). The limited responses of *PtrFBLs* to NAA treatment in *Populus* in this study and in *Arabidopsis* (Parry et al., 2009) indicated that *FBLs* were not very sensitive to auxin. However, a considerably lower expression levels of *PtrFBL1* and *7*, which showed unique expression patterns in the cambial zone (Figure 3), were observed in stems after NAA treatment (Figures 4G,H). This may suggest their specific functions in vascular tissue development.

### Differential stress responses of *PtrFBLs*

**Figure 5:**
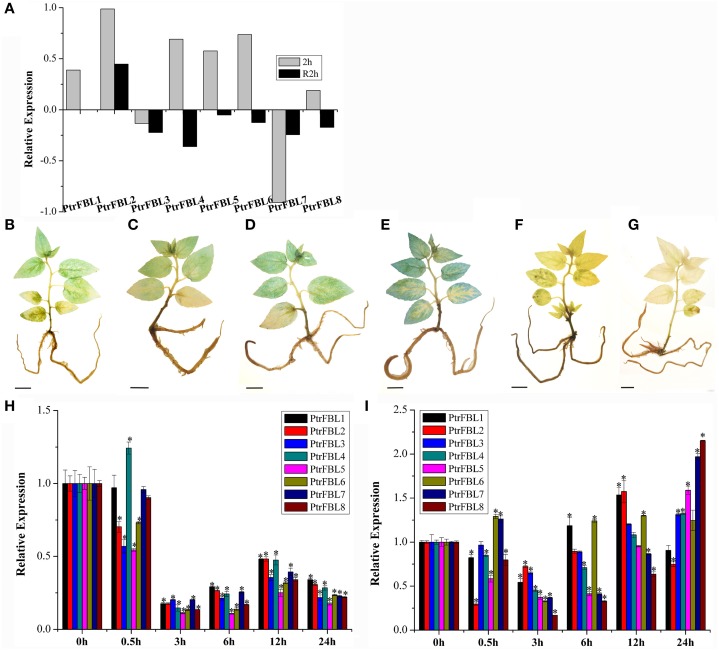
**Expression profiles of ***PtrFBL*** genes under heat and drought treatments. (A)** Heat map showing eight *PtrFBL* genes under heat stress. 2 h: 2 h of 37°C heat treatment; R2h: 2 h recovery after 37°C heat treatment. Endogenous IAA levels expressed in *DR5-GUS* plants under PEG6000 stress **(B–G)**; **(B)** 0 h, **(C)** 0.5 h, **(D)** 3 h, **(E)** 6 h, **(F)** 12 h, and **(G)** 24 h treatments; **(H,I)** Expression profiles of eight *PtrFBL* genes under PEG6000 stress; **(H)** in 84k leaf; **(I)** in 84k stems. Bars, 1 cm **(B–G)**, ^*^indicates significant difference at *P* < 0.05.

Temperature was an important factor that limits the growth, development, and geographical distribution of plants (Nguyen et al., 2009), and high temperatures altered auxin responses by changing auxin synthesis (Franklin et al., 2011). We investigated the expression of the *PtrFBL* genes based on our RNA-seq data (unpublished) from heat-treated seedlings by transferring them from 25 to 37°C and incubating for 2 h. The results showed the different responses of *PtrFBL* members under heat stress (Figure 5A). Previous studies in *Arabidopsis* found that the quadruple mutant *tir*1/*afb*1/*afb*2/*afb*3 exhibited a delayed response but also a strongly reduced hyponastic growth response amplitude after the initiation of the heat treatment (van Zanten et al., 2009). Therefore, FBLs are among the primary factors involved in heat stress response.

Drought treatment with PEG6000 had significant effects on the response of auxin as indicated by the DR5::GUS line (Figures 5B–G). Endogenous auxin increased in the early stage, but decreased in the later stage of the treatment. Relatively low expression levels of all the *PtrFBL* genes in leaves and stems were observed in the first 3 h, but they recovered almost to the same, or higher, levels in the stems. However, they remained low in the leaves 6 h after treatment compared with the untreated plants (Figures 5H,I). The differences in the expression levels of *PtrFBLs* in leaves and stems suggest their regulatory roles in reprogramming plant development to cope with drought stress by lowering the auxin signaling in leaves while strengthening it in stems.

### Over-expression of *PtrFBL1* affected the growth and drought tolerance of transgenic plants

**Figure 6:**
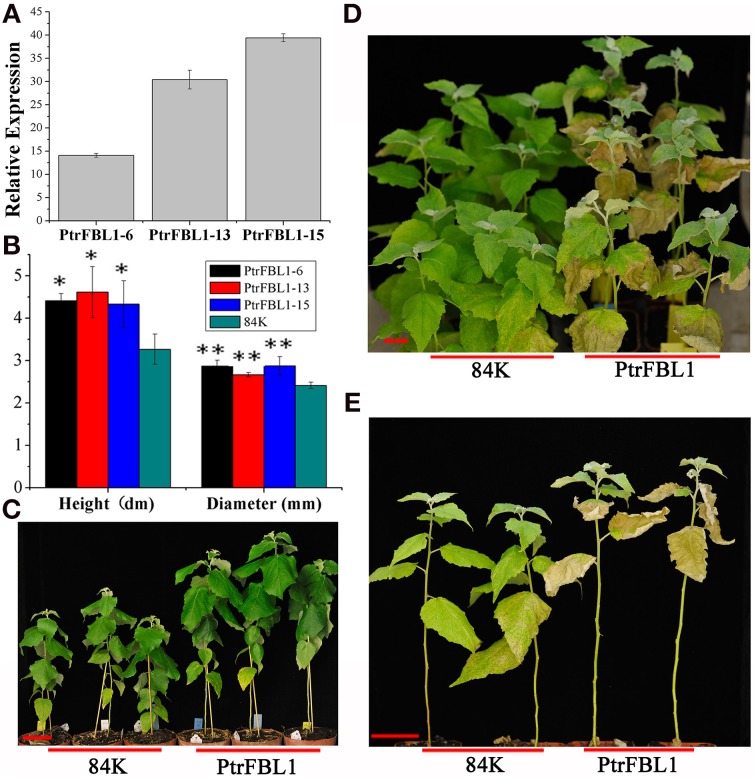
**The growth and drought tolerance of ***PtrFBL1*** over-expressed poplars. (A)** Phenotypic changes in stem growth of over-expressing *PtrFBL1*; **(B)** Relative expression for over-expressing *PtrFBL1*; **(C)** The height and diameters of non- and transgenic lines; **(D,E)** Phenotypic changes of the *PtrFBL1* over-expressed plants after drought treatment for 6 days and the photos were taken 15 days after rewatering. Bars, 5 cm **(A,D,E)**, ^*^indicates significant difference at *P* < 0.05, and ^**^indicates extremely significant difference at *P* < 0.01.

To investigate the role of *PtrFBL1* in stem development, we successfully generated 84K transgenic lines constitutively over-expressing *PtrFBL1* under the control of the *35S* promoter. Three representative transgenic lines with relative expression over 14-fold were used for further analysis (Figures 6A,B). We observed apparent phenotypic changes in height and diameter of the transgenic plants (Figures 6A,C), which were significantly higher than that of the non-transgenic controls (*P* = 0.015 and *P* = 0.006, respectively). However, we found that the transgenic plants exhibited decreased tolerance under drought treatment (Figures 6D,E). RWC was a measure of plant water status and used as a meaningful index of water stress tolerance (Negi et al., 2015). In our study, the RWC values obtained from plants under drought stress showed that the transgenic lines retained less water than the non-transgenic plants (Table 1). The difference was significant for transgenic line *PtrFBL1-13* at 4 days (*P* = 0.011) and for both lines at 6 days (*P* = 0.001) after the deprival of watering. This indicated that PtrFBL1 played a key role in the balance of plant growth and tolerance. Although the roles of TIR1 in many developmental processes in *Arabidopsis* have been well documented (Parry et al., 2009), the effects of its homolog PtrFBL1 in poplar on the stem growth and tolerance were first found in this study.

**Table 1:** **The RWC values of the leaves at 0, 4, and 6 days after the deprival of watering**.

**Line**	**0 day**	**4 days**	**6 days**
PtrFBL1-6	90.113±0.0827	91.237±2.138	89.634±0.213^**^
PtrFBL1-13	90.927±1.173	88.906±1.560^*^	91.220±0.949^**^
Non-transgenic 84k	91.292±1.107	93.680±0.630	93.787±0.639

Value = mean ± SD;

* significance at P ≤ 0.05;

** *significance at P ≤ 0.01*.

The evolutionary features of TIR1/AFB proteins across species revealed considerable conservation of sequence and structure, as well as evolutionary diversity following the genome duplication events and intensive segmental recombination. Expression pattern analysis, however, showed that different members of *PtrFBL*s displayed distinctive expression patterns in different *Populus* organs and tissues. Interestingly, *PtrFBL1* was found to express in vascular tissues abaxial and adaxial to the cambium, while *PtrFBL7* was only observed to be highly expressed in the cambial zone, suggesting their different roles in the development of secondary vascular systems in *Populus*. In addition, most tested genes were changed by exogenous treatments with auxin, heat and drought, and exhibited varying dynamic expression patterns. The further functional analysis of PtrFBL1 has confirmed its role in the stem growth as well as in balancing the growth and tolerance under stress conditions. Our data imply that different *PtrFBL* genes may be involved in particular developmental processes, which deserves further characterization of their roles in the development of woody plants.

## Author contributions

WS carried out all the analyses and drafted the manuscript. YL helped in collecting *Populus* materials. YG performed the NAA experiments, and contributed to the results and discussion sections of the manuscript. HZ helped with transgenic seedling identification, total RNA extraction and RT-PCR analyses. JZ helped with data analyses of bioinformatics, as well as tissue and heat stress experiments. SZ and ML contributed intellectually to all aspects of this research and helped in finalizing the manuscript. All authors read and approved the final manuscript.

### Conflict of interest statement

The authors declare that the research was conducted in the absence of any commercial or financial relationships that could be construed as a potential conflict of interest.

## Supplementary material

Table S1 **Primer sequences for *PtrFBLs* Promoter (P), Subcellular (S), semi-quantitative PCR (RT), and quantitative PCR (qRT)**.

Table S2 ***TIR1* genes families in *Arabidopsis* and *Populus***.

Table S3 **The conserved motifs of *TIR1* proteins in *Arabidopsis* and *Populus***.

Figure S1 **Phylogenetic analysis of the *TIR1* family of genes from *Arabidopsis* and *P. trichocarpa* using full-length nucleotide sequences**.

Figure S2 **Gene duplication relationships in the *TIR1* gene family of *Arabidopsis* and *Populus***. Blue lines represent the other anchor gene pairs in the region, and the red line represents the query locus.

Figure S3 **Genomic localizations of the full-length *PtrFBL* genes**. Eight *PtrFBL* genes were mapped to seven linkage groups (LG). Segmentally duplicated homologous blocks are indicated using the same color. The black lines represents a 10-Mb chromosomal distance.

Figure S4 **Phylogenetic relationships of FBL gene promoters in *P. trichocarpa***. A multiple alignment of the sequences 2.5 kb upstream of the *PtrFBL* genes was executed using ClustalX 2.1 and a phylogenetic tree was constructed using MEGA 5 by the neighbor-joining (NJ) method with 1000 bootstrap replicates.

Figure S5 **Heat map showing the expression of eight *PtrFBL* genes in vegetative tissues (YL, young leaves; ML, mature leaves; PS, primary stem; SS, secondary stem; and R, roots)**.
