# Supplementary material for: A Populus TIR1 gene family survey reveals differential expression patterns and responses to 1-naphthaleneacetic acid and stress treatments
Source: Front Plant Sci. 2015 Sep 10;6:719. doi: 10.3389/fpls.2015.00719 (PMC4585115; doi:10.3389/fpls.2015.00719)
Supplement: Table S1 — Primer sequences for PtrFBLs Promoter (P), Subcellular (S), semi-quantitative PCR (RT), and quantitative PCR (qRT). [file Table1.DOCX]

***Supplementary Material***

**A *Populus TIR1* gene family survey reveals differential expression patterns and responses to 1-naphthaleneacetic acid and stress treatments**

**Wenbo Shu^1,2^, Yingli Liu^2^, Yinghua Guo^2^, Houjun Zhou ^2^, Jin Zhang^2^, Shutang Zhao^2,^* and Mengzhu Lu^1,2^***

^1^  Co-Innovation Center for Sustainable Forestry in Southern China, Nanjing Forestry University, Nanjing 210037, China

^2^ State Key Laboratory of Tree Genetics and Breeding, Research Institute of Forestry, Chinese Academy of Forestry, Beijing 100091, China

*** Correspondence:** Prof.Mengzhu Lu, Co-Innovation Center for Sustainable Forestry in Southern China, Nanjing Forestry University, 159 Longpan Road , Nanjing, Jiangsu, 210037, China. e-mail: [lumz@caf.ac.cn](mailto:lumz@caf.ac.cn); Dr. Shutang Zhao, State Key Laboratory of Tree Genetics and Breeding, Research Institute of Forestry, 8 Xiangshan Road, Beijing, 100091, China. e-mail: Zhaost318@163.com

## Supplementary Tables

**Table S1 Primer sequences used for *PtrFBLs* promoter(P), Subcellular(S) localization, semi-quantitative PCR(**RT**), and quantitative PCR(q**RT**)**

| **Primer** | **Primer sequence** | **Primer** | **Primer sequence** |
| --- | --- | --- | --- |
| **PtrFBL-1RT-F** | GCCTCTTCTTTCAAGGATCG | **PtrFBL-8qRT-F** | GAGAGGCTGGTGACTAGGTC |
| **PtrFBL-1RT-R** | AAGCAGCATCCAATAGCTCA | **PtrFBL-8qRT-R** | TTCACCTTGAGCCACATCCT |
| **PtrFBL-2RT-F** | CATTACAGATCAAGACCCCTG | **PtrUBQ -F** | GTTGATTTTTGCTGGGAAGC |
| **PtrFBL-2RT-R** | CTAGGGCAGTAGAACAATGA | **PtrUBQ -R** | GATCTTGGCCTTCACGTTGT |
| **PtrFBL-3RT-F** | AGCCTGTTGGTGTTCTAGTGGC | **P_PtrFBL-1_-F** | TGTTGTCTGGGTGCGAAAAG |
| **PtrFBL-3RT-R** | TTAACTCCACAAGGAACGTGTC | **P_PtrFBL-1_-R** | ATCTTCTGACCCAGCAGCTT |
| **PtrFBL-4RT-F** | CTCTATAACATCATCTTCTT | **P_PtrFBL-4_-F** | TTGTAGGTTTCTTAGGGAGCTGGAC |
| **PtrFBL-4RT-R** | AACCTAGATCTGGAAACTAG | **P_PtrFBL-4_-R** | ACATTGACCTCACCTTTGAGACATG |
| **PtrFBL-5RT-F** | CAGAGAGTGATTCCAAATCT | **P_PtrFBL-5_-F** | ACTTTTGCCGGCAGATGACT |
| **PtrFBL-5RT-R** | TGACCTTGTTGAGTCAAGAA | **P_PtrFBL-5_-R** | AGTGCAAGTCCTCACCACAG |
| **PtrFBL-6RT-F** | AGACAACATGACAAGCCACAGC | **P_PtrFBL-7_-F** | TTTGGCACTGCTCTGTTATGC |
| **PtrFBL-6RT-R** | CTGCTTCATCCACTTCTGCATA | **P_PtrFBL-7_-R** | TCTGGGATTATGGGATTCCATAG |
| **PtrFBL-7RT-F** | TGCTTTACTCAGTCTATCAG | **S_PtrFBL-1_-F** | ATGTTGAGAAAGGCGAATTC |
| **PtrFBL-7RT-R** | TCATGGGTGATTGAGTTGG | **S_PtrFBL-1_-R** | AGAAAACCTTGACAGAATC |
| **PtrFBL-8RT-F** | CTACTCAGTCTAGTAGACAC | **S_PtrFBL-2_-F** | ATGCCGAACAAGGCGAGTAC |
| **PtrFBL-8RT-R** | GTCGTTGGCGATTGAATCCG | **S_PtrFBL-2_-R** | ACAAAACCTTGACACAGAATC |
| **PtrActin-F** | ACCCTCCAATCCAGACACTG | **S_PtrFBL-3_-F** | ATGAATTATTTCCCTGATGA |
| **PtrActin-R** | TTGCTGACCGTATGAGCAAG | **S_PtrFBL-3_-R** | TAAAGTCCACACGAACTCTGG |
| **PtrFBL-1qRT-F** | CACCATGTGTTGTCTGGGTG | **S_PtrFBL-4_-F** | ATGAATTATTTCCCAGATGA |
| **PtFBL-1qRT-R** | TTCTGACCCAGCAGCTTACA | **S_PtrFBL-4_-R** | TAAAGTCCAAACGAACTCTG |
| **PtFBL-2qRT-F** | AACGACAAAGACAGGAACGC | **S_PtrFBL-5_-F** | ATGGGTCCAAATCCAAAGATG |
| **PtrFBL-2qRT-R** | AGCTCCACCGATCTTAGCTC | **S_PtrFBL-5_-R** | TAAACCTGAGAGAGTGAGTAC |
| **PtrFBL-3qRT-F** | TGGTTGCAAGAAACTTCGCA | **S_PtrFBL-6_-F** | ATGGATTCGAATCCAAAGATG |
| **PtrFBL-3qRT-R** | GAGCCTTGGCATCTTCTTCG | **S_PtrFBL-6_-R** | TAAACCTGAGAGAGTCAGTAC |
| **PtrFBL-4qRT-F** | TTGTAGGTTTCTTAGGGAGCTGGAC | **S_PtrFBL-7_-F** | ATGATCACCAACAAAAAGCCTAGA |
| **PtrFBL-4qRT-R** | CATTGACCTCACCTTTGAGACATG | **S_PtrFBL-7_-R** | TTTATGATCAAATGAGTTCGAAAAG |
| **PtrFBL-5qRT-F** | ACTTTTGCCGGCAGATGACT | **S_PtrFBL-8_-F** | ATGATCACCAACAAAAAGCCTAGATC |
| **PtrFBL-5qRT-R** | AGTGCAAGTCCTCACCACAG | **S_PtrFBL-8_-R** | CAAGATGGAAACAAATCGTGGCACA |
| **PtrFBL-6qRT-F** | AGTGCATGCTGGAAGGTTGT | **PtrActin-F** | ACCCTCCAATCCAGACACTG |
| **PtrFBL-6qRT-R** | ACCCGACAGCCATTCATTGT | **PtrActin-R** | TTGCTGACCGTATGAGCAAG |
| **PtrFBL-7qRT-F** | CATGAAGATAATGTGGACGTGGATG |  |  |
| **PtrFBL-7qRT-R** | TCAAACTTTCTCTTGCAAGGATCAC |  |  |

**Table S2*TIR1* genes families in *Arabidopsis*, and *Populus***

| **Gene name** | **Locus** | **Genomic position** | **Mol.Wt (kDa), Length(aa),pI** | **Gene length, ORF, introns** | **WOLFPSORT predictions*** |
| --- | --- | --- | --- | --- | --- |
| **At TIR1** | At3g62980 | Chr3: 23273116-23276375（-） | 66.79,594,6.99 | 2703,1785,2 | N:5, Ch:4,C:2,Cy:2 |
| **At AFB1** | At4g03190 | Chr4: 1404887-1407139（-） | 65.65,585,8.12 | 1950,1758,2 | Ch:7, N:5, Cy: 2 |
| **At AFB2** | At3g26810 | Chr3: 9867845-9870640（+） | 64.60,575,7.17 | 2123,1728,2 | N: 7, Cy: 4, Ch: 3 |
| **At AFB3** | At1g12820 | Chr1: 4368760-4371293（-） | 64.91,577,6.89 | 2067,1734,2 | N: 6, Ch: 4,Cy: 3 |
| **At AFB4** | At4g24390 | Chr4: 12613590-12616117（-） | 69.49,623,6.31 | 2058,1872,2 | N:8.5,CN:6,Cy:2.5,Ch: 2 |
| **At AFB5** | At5g49980 | Chr5: 20333964-20336665（-） | 69.32,619,5.14 | 2112,1860,2 | N:7.5,CN: 4.5,Ch:4,p: 1 |
| **At COL1** | At2G39940 | Chr2: 16672493-16675748（-） | 67.67,592,6.06 | 2639,1779,2 | Cy: 11, N: 1,M: 1 |
| **PtrFBL1** | Potri.014G134800 | Chr14:10256941-10259719（-） | 65.15,584,7.35 | 2396,1755,2 | Ch:7,Cy: 3,N: 2, P: 1 |
| **PtrFBL2** | Potri.002G207800 | Chr02: 17456876-17460235（+） | 65.28,584,5.60 | 2499,1755,2 | N:5.5,CN:5,Ch:4, Cy: 3.5 |
| **PtrFBL3** | Potri.001G323100 | Chr01: 32724931-32728893（+） | 64.16,571,6.90 | 2851,1716,2 | N:6,Cy: 4,Ch: 2,P: 1 |
| **PtrFBL4** | Potri.017G061600 | Chr17: 5741704-5745358（+） | 64.08,571,7.13 | 2792,1716,2 | Cy: 6, N: 5, ch: 3 |
| **PtrFBL5** | Potri.004G033900 | Chr04: 2486214-2494271（-） | 65.61,585,5.96 | 4366,1758,2 | N:10.5,CN:6.5,Cy: 1.5 |
| **PtFBL6** | Potri.011G042400 | Chr11: 3589019 - 3595391（-） | 65.64,585,5.90 | 4663,1758,2 | N:10.5,CN:6, Ch:1,M: 1 |
| **PtrFBL7** | Potri.005G159300 | Chr05: 16044862-16048464（+） | 70.99,635,5.60 | 2847,2172,3 | Ch: 5, ER: 4,N: 3, M: 1 |
| **PtrFBL8** | Potri.002G102700 | Chr02: 7423681-7427320（-） | 70.94,635,5.47 | 2488,1908,2 | N:8, Ch: 3, Cy: 2 |

**Table S3 The conserved motifs of TIR1 proteins in *Arabidopsis*, and *Populus*.**

| **Motif** | **Width (aa)** | **Sites** | **LLR (Log Likelihood Ratio)** | **E-value** | **Regular expressionSequence(aa)** |
| --- | --- | --- | --- | --- | --- |
| 1 | 70 | 15 | 2430 | 2.7e-629 | N[AS][VA]SLVC[KR]SWY[KER][IV]E[RSA]W[STC]R[KQS][KEHR]VF[IV]GNCY[AS][VI]SP[EA]R[VL][IT]RRFP[RE][LIV][RK]S[VL][TE]LKGKP[HR]FADFNL[VM]P[DPEH][NGD]WG[GA][FY][VF][YAH]PW[IV] |
| 2 | 70 | 14 | 2325 | 2.4e-604 | [VA]F[EL]YIG[MKT]Y[AG][KE][KQ][LV][ER][MT]LS[VI]AFAGD[ST]D[KLM]G[LM][HQ][YH][VL]L[ENS]GC[PKE][KR]L[RQ]KLEIRD[CS]PFG[DN][AK]ALL[SA][GDN][ALV][HAG][KR][YL][EY][TN]MR[SF]LWMS[SA]C |
| 3 | 70 | 14 | 2310 | 7.4e-596 | GL[VQ][AS][IV]SEGC[RP]KL[HEQ]S[IV]LYFC[QR][QR]MTNAA[LV][IV][TA][IVM][AS]KN[CR]P[ND][FLM]T[RCV]FRLCI[MIL][EG][PR]H[AKQ]PD[HY][VL]T[NGL][EQ]P[LM]DEGFGAIV[EK]NCK |
| 4 | 70 | 14 | 2151 | 7.2e-512 | KRMV[VI][TS]D[ED][SCD]L[EA][LF][LI][AS][REK]SF[PK][NG]FK[VES]LVL[VS][SC]C[ED]GF[SGT]T[DS]G[LI]A[AI][IV]A[AS][NK]CR[NQ]L[RK][EV]LDL[QRI]E[SN][ED][VI][DE]D[DH][SER][GV][DQH]W[LI]SCFP[DE][TS] |
| 5 | 59 | 21 | 2003 | 6.5e-392 | [LT][NS][FL][AN]CLYAE[IV][NS]FAALERLV[SA]R[CS]P[NS]L[KQ]SL[RKW][LV][NL][RD][AS][VI][PS][LD][ED]GL[QA][RA]L[LAM][AV][CR][AC][PK]QL[TV]EL[GR][TV][GF][SP][FY]S[PA] |
| 6 | 42 | 21 | 1407 | 9.4E-255 | QPEQ[YG]S[EDK]LAS[AL][FIL]RxCKSL[RQ][SRC]L[SW]G[FL][DW]E[VIA]G[PD]K[YG]L[PE][AV]V[YA][PS]VC[KP]NL |
| 7 | 38 | 13 | 945 | 9E-162 | [END][VEN][RE]G[MP][DP][ED][DS]R[DP][ED][RSD][CQS][KPY]V[ED][KT][LV][YF][LI]YR[TS][VL][AV]G[PR]RKD[AM]P[PEG]FVW[TI][LM] |
| 8 | 30 | 6 | 386 | 2E-32 | x[EP][EFS][LY][DS][AS][PT][RY][PS][DS][HQ][VF][LP][ED][NE]VLE[NR]VL[QSW][FL]L[TK]S[RH][KC]DR |
| 9 | 30 | 8 | 360 | 7E+05 | [DGM]I[TS][ND][EK][GK][LP][ER]S[ISV][AD][TR]D[CS][KNR][NY][LIM]RD[LDV]R[TM]EM[SF][DE][DFP][DS][ED]R |
| 10 | 41 | 6 | 342 | 3.3E+18 | KxxDNLxVDx[GP][GY]xLx[EL]xI[GS]xx[LE]MxxMxV[FL]xx[IK][GL]xxEGLx[SY]I |
| 11 | 30 | 6 | 251 | 1.5E+19 | [KL][DK][ED][ET]E[GD][EK][KV][EK][EDK]V[DE]E[EL]KE[KE][FKE][EK][KG][KL][KC]K[KV]IK[ED]V[SL][HG] |
