# Supplementary figures and images for: A Populus TIR1 gene family survey reveals differential expression patterns and responses to 1-naphthaleneacetic acid and stress treatments
Source: Front Plant Sci. 2015 Sep 10;6:719. doi: 10.3389/fpls.2015.00719 (PMC4585115; doi:10.3389/fpls.2015.00719)

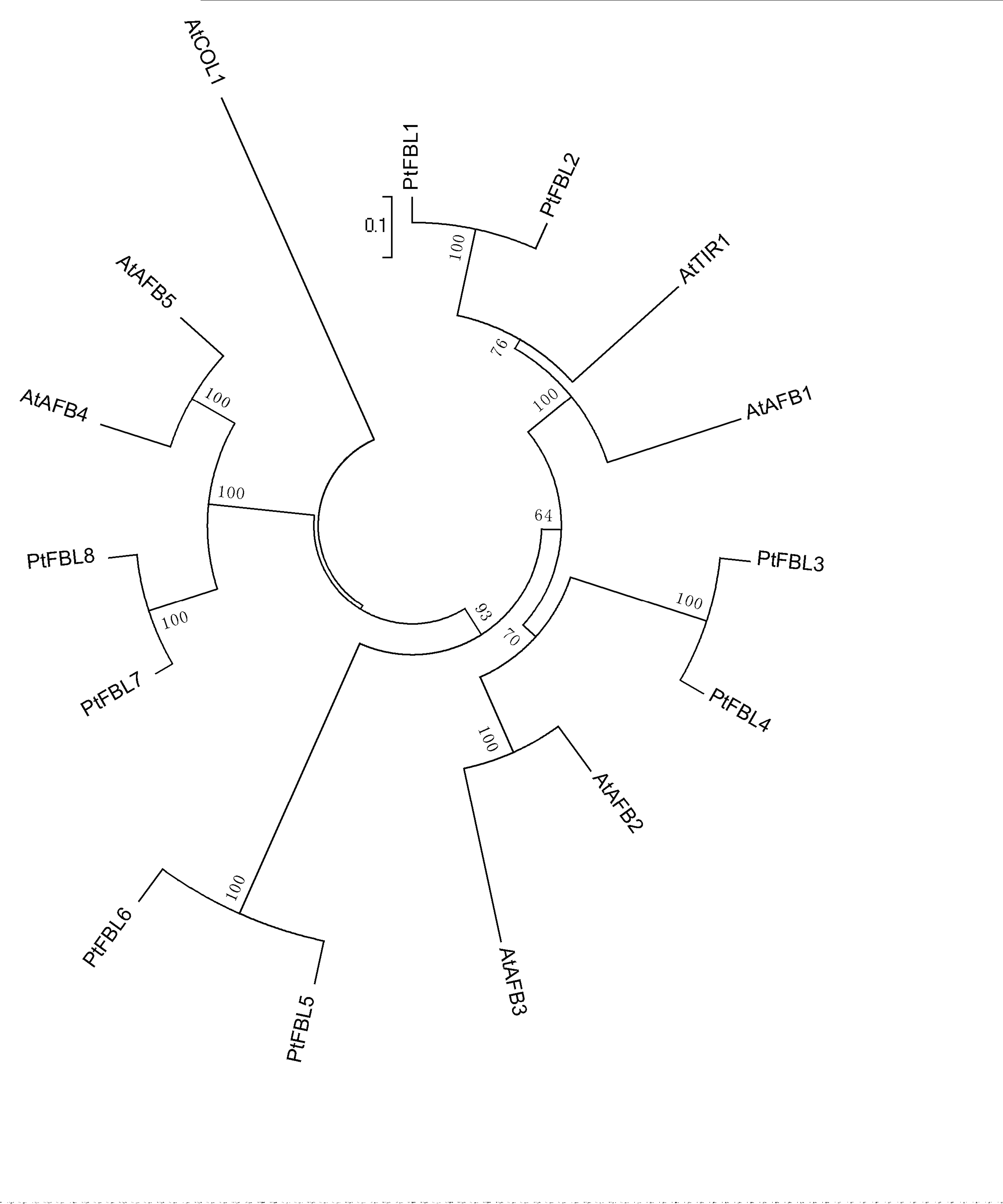

Supplement: Figure S1 — Phylogenetic analysis of the TIR1 family of genes from Arabidopsis and P. trichocarpa using full-length nucleotide sequences. [file Image1.TIF]

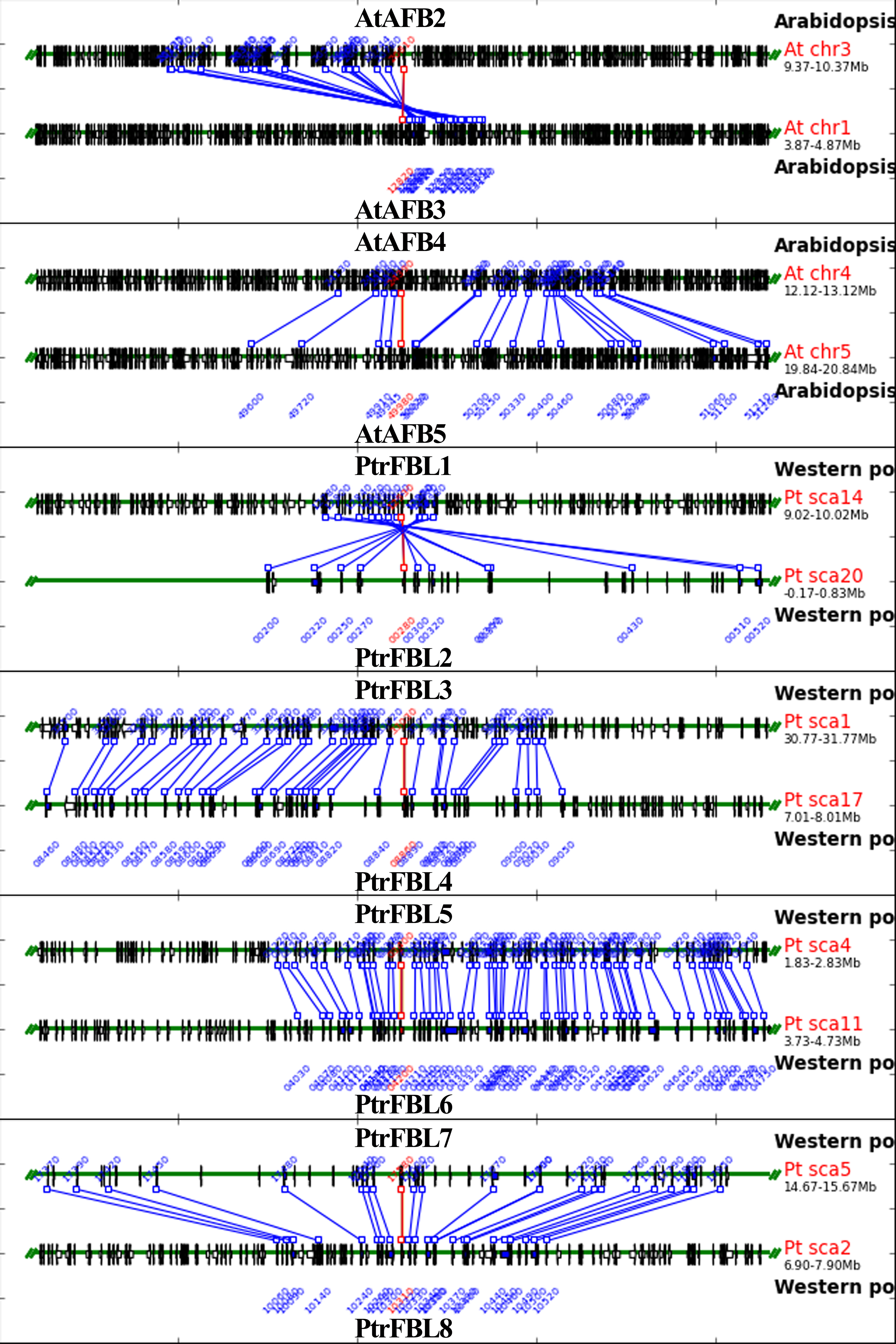

Supplement: Figure S2 — Gene duplication relationships in the TIR1 gene family of Arabidopsis and Populus. Blue lines represent the other anchor gene pairs in the region, and the red line represents the query locus. [file Image2.JPEG]

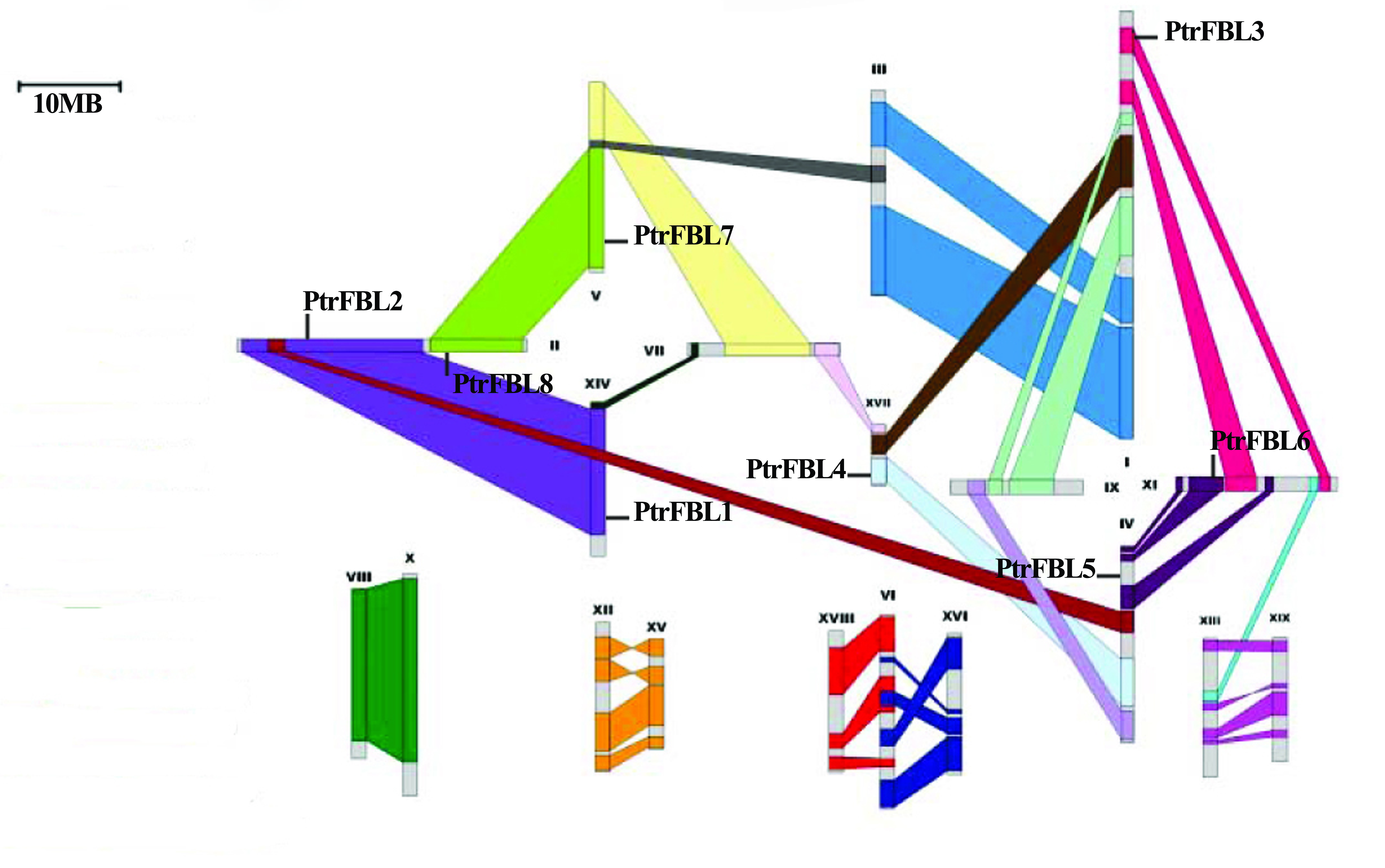

Supplement: Figure S3 — Genomic localizations of the full-length PtrFBL genes. Eight PtrFBL genes were mapped to seven linkage groups (LG). Segmentally duplicated homologous blocks are indicated using the same color. The black lines represents a 10-Mb chromosomal distance. [file Image3.JPEG]

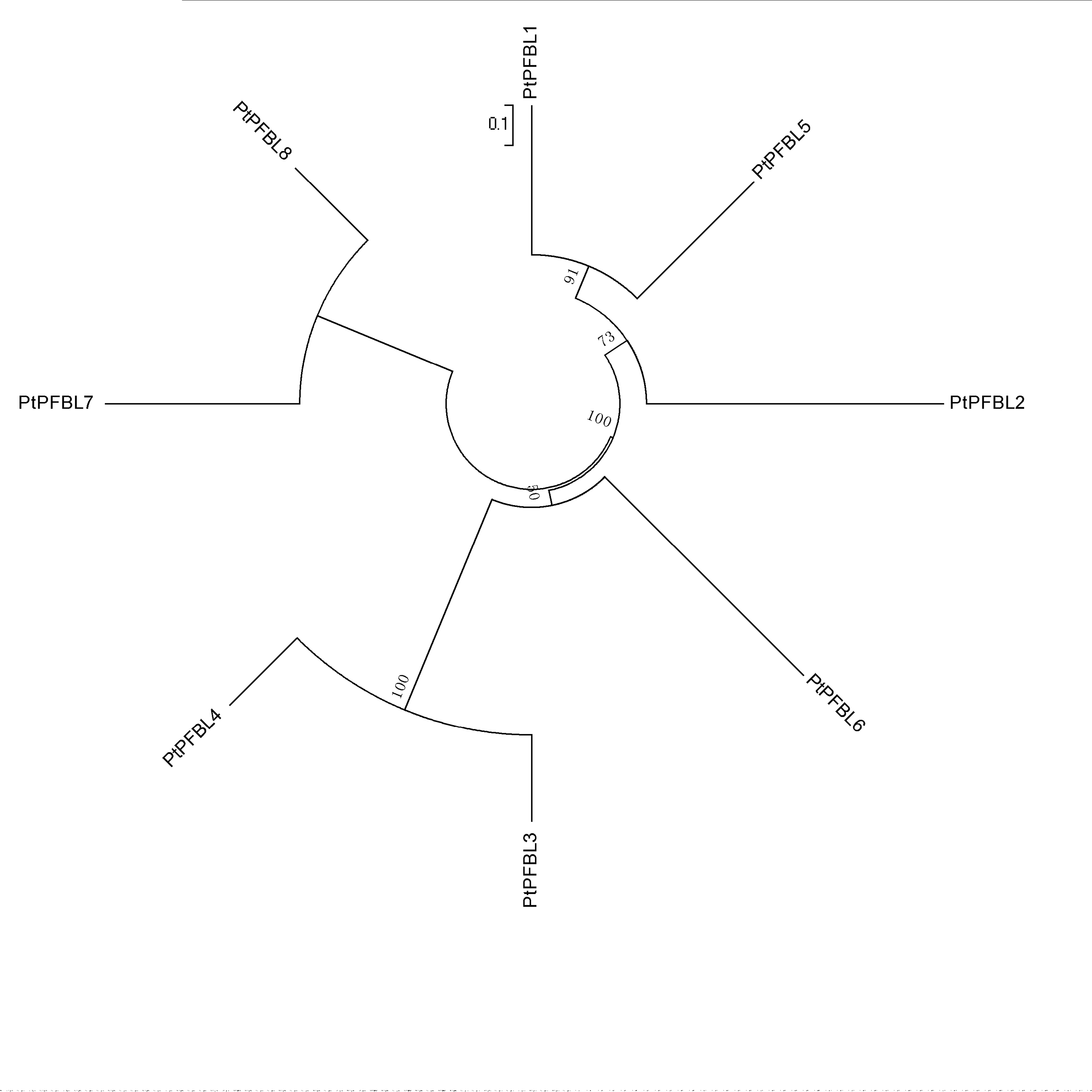

Supplement: Figure S4 — Phylogenetic relationships of FBL gene promoters in P. trichocarpa. A multiple alignment of the sequences 2.5 kb upstream of the PtrFBL genes was executed using ClustalX 2.1 and a phylogenetic tree was constructed using MEGA 5 by the neighbor-joining (NJ) method with 1000 bootstrap replicates. [file Image4.TIF]

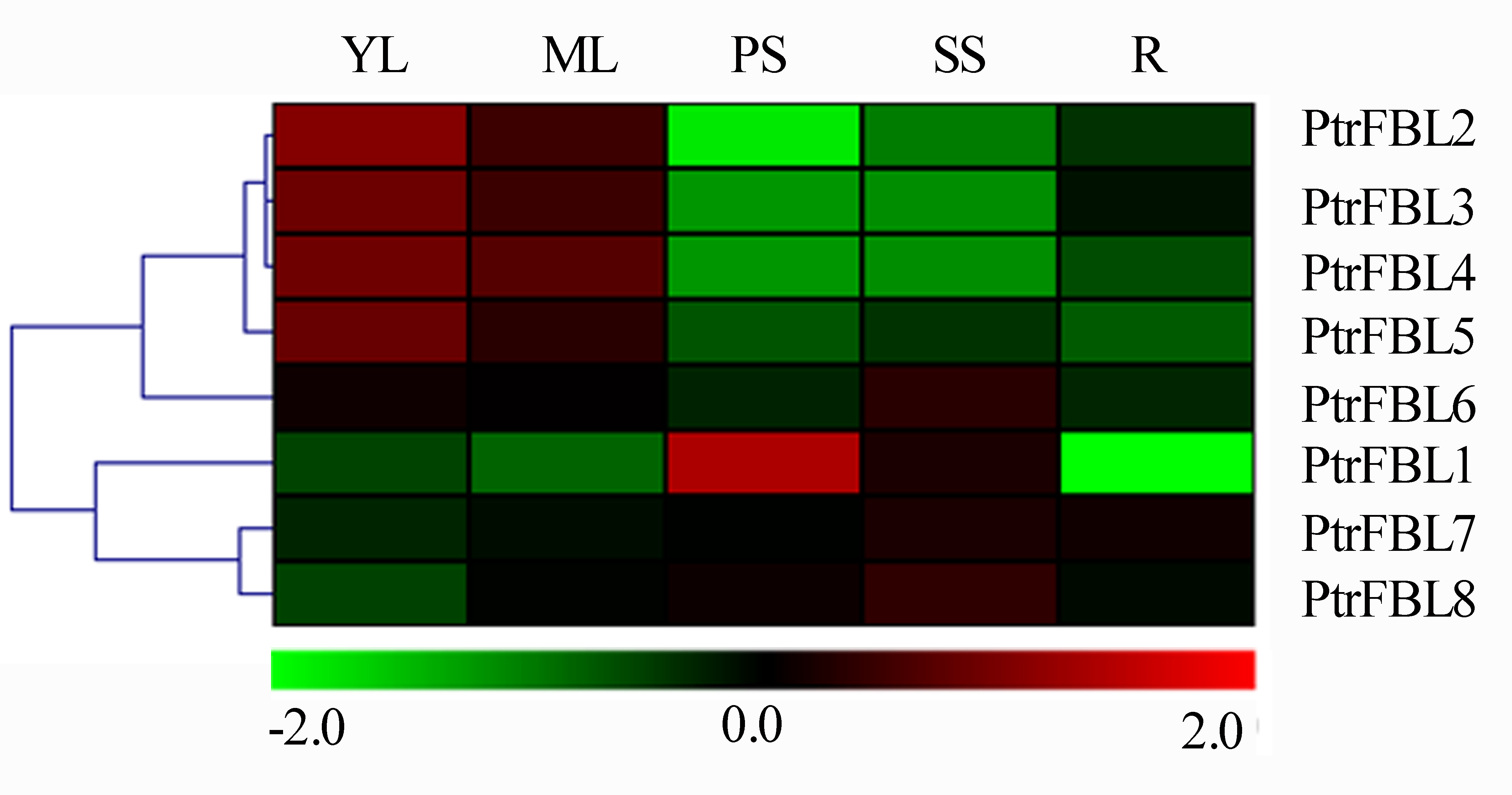

Supplement: Figure S5 — Heat map showing the expression of eight PtrFBL genes in vegetative tissues (YL, young leaves; ML, mature leaves; PS, primary stem; SS, secondary stem; and R, roots). [file Image5.JPEG]
